# IMPACT OF HUSBAND’S DEMENTIA: INNOVATIVE METHODS TO STUDY HEALTH PROFILES AND CO-TRAJECTORIES BETWEEN COUPLES

**DOI:** 10.1093/geroni/igad104.0900

**Published:** 2023-12-21

**Authors:** Yan Zhang, Yiyang Yuan, Maria Roche-Dean, Richard Gonzalez, Irving Vega

**Affiliations:** East Carolina University , Greenville, North Carolina, United States; University of Massachusetts Chan Medical School, Worcester, Massachusetts, United States; Western Michigan University, Kalamazoo, Michigan, United States; University of Michigan, Ann Arbor, Michigan, United States; Michigan State University, Grand Rapids, Michigan, United States

## Abstract

Traditional life course studies often focus on how one predictor influences one health outcome that may not be able to capture the interdependence of two or more health trajectories very well.

Objectives: (1) demonstrate an innovative method to create a 3-dimensional health profile cube that presents physical, mental, and cognitive health and the changing trajectories over time; (2) display how a woman’s health profile may change due to the onset of their male partner’s dementia; and (3) visualize how couple’s co-trajectories of health profiles may vary by their age, race/ethnicity, and household wealth.

Methods: We focus on married and partnered couples, drawing longitudinal data from the Health and Retirement Study (2000-2016; n=3,578). Physical, mental, and cognitive health were respectively measured by functional limitation, depression symptoms (CESD-II), and modified version of the Telephone Interview for Cognitive Status (TICS). We use vector autoregression (VAR) models to analyze these multivariate paths in the same visual representation as the original data.

Results: The approach provides both an analytic framework and a visualization tool that depicts data and model in the same spatial representation to permit assessment of model fit and model comparison. This study advances the traditional life course studies by representing underlying processes as a multidimensional time vector of health outcomes, which better reflect the interdependent co-trajectories of health outcomes and the essence of the life course conceptual framework.

Discussion: This paper provides a blueprint for studying complex health profiles or trajectories.

